# Ferroptosis: A Trigger of Proinflammatory State Progression to Immunogenicity in Necroinflammatory Disease

**DOI:** 10.3389/fimmu.2021.701163

**Published:** 2021-08-18

**Authors:** Jing-yan Li, Yong-ming Yao, Ying-ping Tian

**Affiliations:** ^1^Department of Emergency, The Second Hospital of Hebei Medical University, Shijiazhuang, China; ^2^Translational Medicine Research Center, Medical Innovation Research Division and Fourth Medical Center of the Chinese PLA General Hospital, Beijing, China

**Keywords:** ferroptosis, necroinflammatory diseases, inflammatory response, immunogenicity, immune cell

## Abstract

Until recently, necrosis is generally regarded as traumatic cell death due to mechanical shear stress or other physicochemical factors, while apoptosis is commonly thought to be programmed cell death, which is silent to immunological response. Actually, multiple modalities of cell death are programmed to maintain systematic immunity. Programmed necrosis, such as necrosis, pyroptosis, and ferroptosis, are inherently more immunogenic than apoptosis. Programmed necrosis leads to the release of inflammatory cytokines, defined as danger-associated molecular patterns (DAMPs), resulting in a necroinflammatory response, which can drive the proinflammatory state under certain biological circumstances. Ferroptosis as a newly discovered non-apoptotic form of cell death, is characterized by excessive lipid peroxidation and overload iron, which occurs in cancer, neurodegeneration, immune and inflammatory diseases, as well as ischemia/reperfusion (I/R) injury. It is triggered by a surplus of reactive oxygen species (ROS) induced in an imbalanced redox reaction due to the decrease in glutathione synthesis and inaction of enzyme glutathione peroxidase 4 (GPX4). Ferroptosis is considered as a potential therapeutic and molecular target for the treatment of necroinflammatory disease, and further investigation into the underlying pathophysiological characteristics and molecular mechanisms implicated may lay the foundations for an interventional therapeutic strategy. This review aims to demonstrate the key roles of ferroptosis in the development of necroinflammatory diseases, the major regulatory mechanisms involved, and its potential as a therapeutic target.

## Introduction

**Figure 1:**
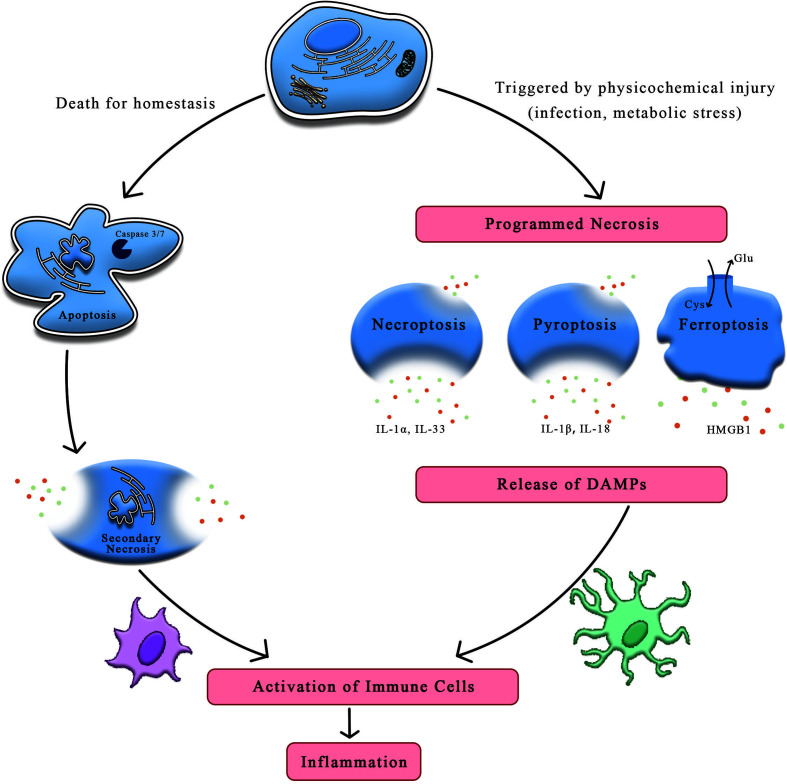
The distinctions between apoptosis and necrosis. On account of the stimuli and context, cells can undergo apoptosis and necrosis. Apoptosis is thought to be programmed cell death relating to homeostasis while necrosis is induced by mechanical shear stress or other physicochemical factors including infection and oxidation, etc. Upon the stimulation of damaged signals, cells trigger programmed necrosis, such as necroptosis, pyroptosis and ferroptosis. Cells suffering from stress may release immunogenic molecules called DAMPs, which could initiate the systematic immune response against detrimental substances, ultimately leading to inflammation. HMGB1, high mobility group box-1 protein; DAMPs, danger-associated molecular patterns.

Apoptosis, regarded to occur only in one form of programmed cell death, is deemed to play a part in homeostasis and host defense. This form yields cell death in a genetically regulated way that performs an induced impact on the adjacent cells. While another different form of necrosis, considered as a type of cell death in the setting of physicochemical stimulation, can be dictated by a special molecular pathway that releases intracellular contents to induct inflammatory response (1). Compared to the formation of apoptotic bodies and membrane packaging during apoptosis, necrosis is characterized by cellular swelling, membrane permeabilization, and even release of cellular contents (2). Although conspicuous feature of these cell death processes may transform into differently immunogenical levels, impairment of scavenging function in apoptotic cells can result in necrosis, inducing the onset of inflammation (3). It is proved by accumulating evidences that cells indeed undergo programmed necrotic processed, such as necroptosis, pyroptosis, ferroptosis, and NET osis (**Figure 1**).

Necroinflammation, defined as the cascade connection of innate and adaptive immune responses to necroptotic cell death, might be regulated by particular signaling mechanisms such as necroptosis, ferroptosis, and pyroptosis. Cells suffering from oxidative stress may release immunogenic molecules, which trigger the systematic immune response against detrimental substances, ultimately leading to necrotic cell death in a physiological or pathophysiological state. Therefore, immune cell should be dependent on precisely discriminative mechanism to distinguish between the diverse forms of cell death, and concurrently detect signaling molecular transmitted by dying cells for activating immune system. As to date, evidences prove immune response to be affected *via* ferroptosis during programmed necroinflammatory process. A better understanding of ferroptosis as a form of necrosis, could lead to the pharmacological prevention in necroinflammatory disease.

Ferroptosis, characterized by iron-dependent lipid peroxidation, and triggered by a particular small-molecule inducer, is inducted by unique and precise mechanisms. In fact, superimposed lipid peroxidation has been confirmed to be the central part of ferroptosis. A long-chain-fatty-acidacetyl-coenzyme A synthase 4 (ACSL4) (4), which is an activator of the lipoxygenase-dependent signaling pathway, involves in ferroptosis initiation, and another antioxidant enzyme glutathione peroxidase 4 (GPX4) (5) subsequently triggers ferroptosis under the reduction/oxidation imbalanced status. The specific necrotic signaling pathway of ferroptosis may produce pathogenic cytokines peroxides that impair the immune response *via* activating immune cells. In addition, ferroptosis may upregulate subcellular structures such as hazardous peroxisomes on the surface of fractured organelles or ruptured mitochondria. More studies suggest that ferroptosis-related cell death has a potential link to necroinflammatory disease. Hence, further exploration in ferroptosis enhancing systematically proinflammatory state of the immune response might potentially target for novel mechanisms and therapies.

## Ferroptosis and Regulatory Mechanisms

**Figure 2:**
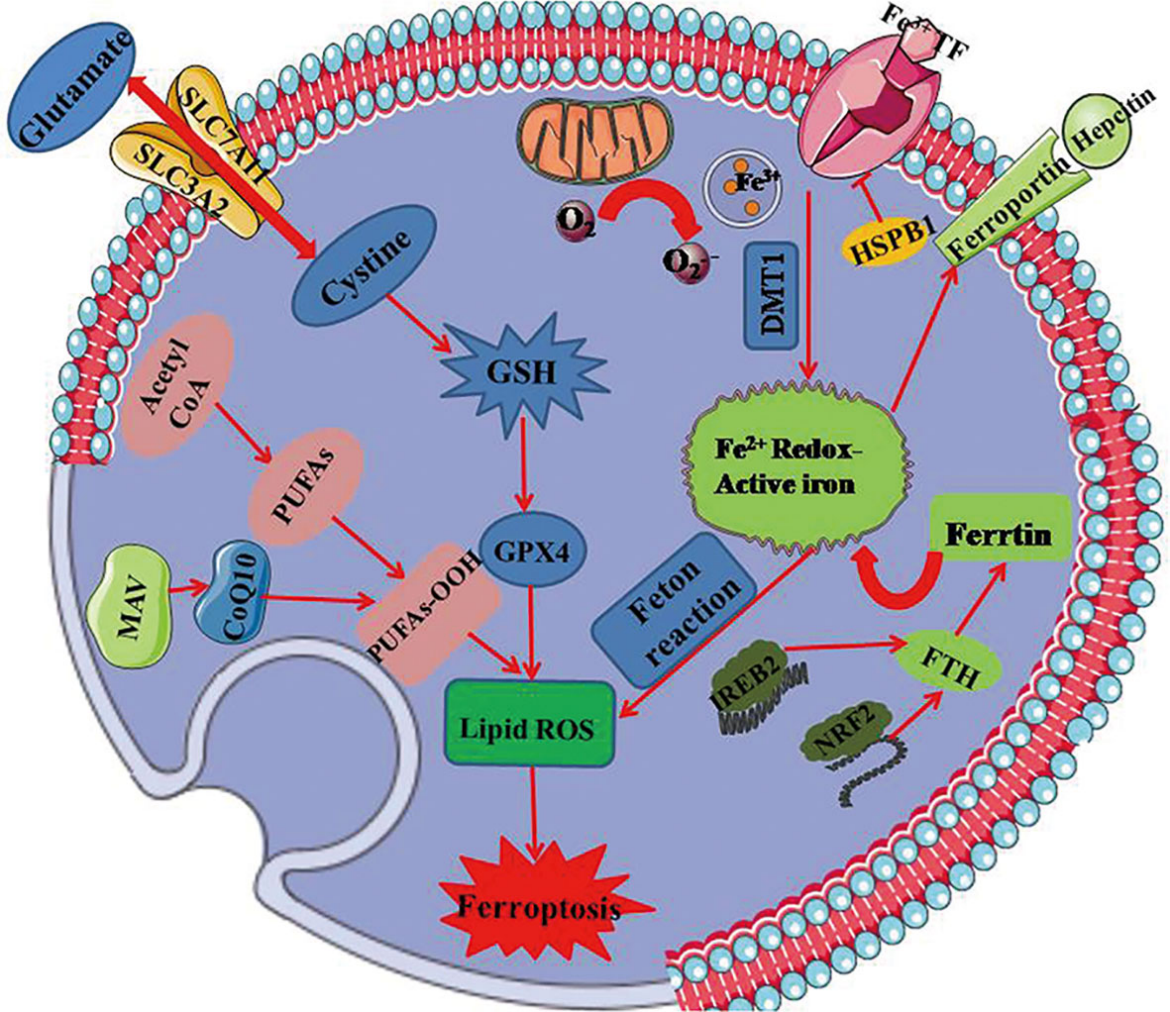
The mainly regulated mechanism underlying ferroptosis. GPX4 regarded as the key regulator in ferroptosis relies on the biosynthesis of GSH. It produces an antioxidative effect on ferroptotic process, and is regulated by MAV signaling pathway. Xc- system that is composed of SLC7A11 and SLC3A2 regulates ferroptosis together with glutathione metabolic pathway by exchanging glutamate and cystine at 1:1 ratio. Ferroptosis is dependent on overload iron that ascribes to peroxides and divalent ferrous salts produced by fenton reaction. Iron can be transported from extracellular to intracellular in virtue of transferring protein. Mitochondria as the essential organ involving in ferroptosis, contains six ferroptosis-related genes and releases ferroptosis-induced lipid peroxides through the electron-transporting chain. PUFAs, polyunsaturated fatty acids; GSH, glutathione; GPX4, glutathione peroxidase 4; DMT1, divalent metal transporter 1; HSPB1, heat shock protein B1; FTH, ferritin heavy polypeptide; IREB2, iron response element binding protein 2; NRF2, nuclear factor erythroid-2-related factor 2; SLC7A11, transmembrane protein transporter vector family 7 member 11; SLC3A2, single-channel transmembrane regulatory protein solute carrier family 3 member2.

Ferroptosis differs from other traditional forms of cell death in terms of initiative factor, dying cell morphology, regulatory pathway, as well as biological induction and inhibition (5, 6). It is notable that ferroptosis is a mitochondria-dependent type of cell death with the features in mitochondrial morphology including reduced mitochondrial volume, increased inner membrane density, rupture of the outer mitochondrial membrane and mitochondria cristae dysfunction (7). Moreover, the key mechanisms involved in ferroptosis depend on the metabolism of polyunsaturated fatty acid and the modulation of phospholipidome, especially the nonenzymatic lipoxygenase-mediated lipid peroxidation leading to the destruction of the lipid bilayer. Thus, ferroptosis occurs along with glutathione overconsumption, inhibition of glutathione synthesis and reduction of GPX4 activity when the redox reaction is disorganized. Up to date, as increasingly advanced understanding of ferroptosis in various domains, the signaling cascades including System Xc-, GPX4, MVA, and heat shock factor (HSF)1-HSPB1 (8) appear to be more clear. The major signaling pathways of ferroptosis are summarized in **Figure 2**.

### Glutathione Metabolic Pathway

The activity of lipid repair enzyme called GPX4 which exerts an antioxidative effect on ferroptotic process depends on the biosynthesis of glutathione (GSH) (5), and is identified as a key regulatory factor in ferroptosis. Intracellular depletion of GSH leads to GPX4 inactivation and lipid peroxidation accumulation, eventually resulting in ferroptosis (9). In particular, GPX4 is specifically targeted by the endogenous ferroptosis-inducing agent of RSL3 (10), which catalyzes GSH-dependent reduction of hydroperoxides to lipid alcohols System Xc- composed of a transmembrane protein transporter solute carrier family 7 member 11 (SLC7A11) and a single-pass transmembrane regulatory protein solute carrier family 3 member 2 (SLC3A2), regulates ferroptosis together with glutathione metabolic pathway by exchanging glutamate and cystine at 1:1 ratio (11). Inhibition of system Xc- causes the depletion of intracellular cysteine, restricting the synthesis of glutathione, and triggering oxidative stress, and then the antioxidant enzyme GPX4 is impaired, which finally initiates ferroptosis. Moreover, increasing evidences support the hypothesis that trans-sulfuration as another regulator of ferroptosis, is the major source of compensatory for cysteine depletion and further inhibits erastin-induced ferroptosis (12). Therefore, GPX4 synthesis-related and system Xc- function-related pathway are essential in ferroptotic regulation.

### Lipid Peroxidation Pathway

Researchers have discovered that ferroptosis is preferentially accompanies by lipid peroxidation including polyphosphorylated phosphatidylethanolamine (PE) containing polyunsaturated fatty acids (PUFAs) (13). ACSL4 is considered as the key enzyme to regulate lipid oxidative response and accelerate ferroptosis by generating oxidized PE in oxygenation localize, catalyzing adrenaline (AdA) to generate AdA acyl Co-A, which is esterified to AdA-PE (14). A mass of malondialdehydes is produced by AdA-PE oxidation and ultimately leads to ferroptosis. Expression of ACSL4 is regulated by certain molecules, such as special protein 1 (Sp1) (15), a transcription factor that upregulates ACSL4 transcription to promote ferroptosis. Inhibiting the activity of ACSL4 can block AdA esterificating into PE, which reduces susceptibility of mouse embryonic fibroblasts Pfa 1 cells to ferroptosis (13). These studies indicate that lipid peroxidation is the key step in ferroptosis.

### NADPH-FSP1-CoQ10 Pathway

It has been demonstrated by Doll and colleagues that overexpression of apoptosis-inducing factor mitochondria-associated 2 (AIFM2, also named as FSP1) (16) is capable of reversing GPX4 suppression-induced ferroptosis, which proves FSP1 to be ferroptotic inhibitor independent on GPX4 mechanism. The N-terminus of FSP1 is representative for its structural domain called myristoylation with function of lipid modification, which promotes FSP1 locating on plasmalemma and reduces sensitivity of cells to ferroptosis (16). A previous study verified FSP1 to be an nicotinamide-adenine dinucleotide phosphate- (NADP-) dependent coenzyme Q (CoQ) oxidoreductase, which is an electronic carrier and acts as lipidsoluble antioxidant (17). The recent studies demonstrate that FSP1 is paralleled with GPX4 to suppress ferroptosis by directly regulating the nonmitochondrial CoQ_10_ antioxidant system (18). Hence, inhibition of FSP1 combined with GPX4 might provide a more effectively targeting strategy for ferroptosis-associated diseases.

### Iron Metabolism Pathway

Both process and development of ferroptosis relies on overload iron that ascribe to peroxides and divalent ferrous salts produced by fenton reaction. When intracellular iron homeostasis is disordered, nuclear receptor coactivator 4 (NCOA4) (19) mediated ferritinophagy leads to disfunction of transferrin, which ultimately enhances the production of oxygen centered free radicals to induce ferroptosis. The key encoder named iron responsive element-binding protein 2 (IREB2) (7) is responsible for regulating iron metabolism, and studies have revealed that silencing-expressed IREB2 might perform an impact on not only iron transportation but also genes expression of transferrins (5, 7, 8). Moreover, upregulation of autophagy-associated protein expression activates ferroptosis, and inhibition of autophagy-related 5 (Atg5) as well as autophagy-related 7 (Atg7) genes shows a suppressive activity on ferroptosis (20).

### Other Molecular Related Signaling Pathway

In addition to the aforementioned signaling pathway, the nuclear factor erythroid 2-related factor 2 (Nrf2) (8, 21) involves in the regulation of ferroptosis due to its antioxidant function. When cells confront the normoxic setting, Nrf2 is united with Kelch-like ECH-associated protein 1 (Keap 1) to maintain an inactivated state by ubiquitylation in the proteasome, while Nrf2 is released from the conjugated Keap1 protein to transsituate to the nucleus under the oxidative stress (21). In 2016, Sun and his team demonstrated p62-Keap1-Nrf2 signaling pathway performed an antioxidative effect on the regulation of ferroptosis in hepatoma carcinoma cells, depended on the mechanism that p62 as an autophagy receptor could locate on cells to activate Nrf2 by devitalization of Keap1 (22). Nrf2-inhibited ferroptosis is also associated with the mediation of NQO1 (22), home oxygenase-1 (HO-1) (8), and ferritin heavy chain (FTH1) (5, 7), which shows a crosstalk between ferroptosis and autophagy. P53 is reported to mediate ferroptotic signaling pathway through down-regulating SLC7A11 expression to inhibit Xc- system. It is found that proliferation of ROS after activated P53 reduces antioxidant efficacy eventually contributing to ferroptosis, which is reversed by the treatment ferrostatin-1 (Fer-1) (23). Thus, P53 performs an essential impact on ROS-related metabolic signaling pathway of ferroptosis.

## The Relationship Between Ferroptosis and Necroinflammatory Response

Inflammation, generally in response to pathogen or tissue injury, is typically described as a complex biological response (24). The molecular mechanism of inflammation firstly proposed by Charles Janeway, states that the immune system can discriminate between self (healthy tissues) and non-self (invasivepathogens). Accordingly, pathogenic molecules, defined as pathogen-associated molecular patterns (PAMPs) are recognized by pattern recognition receptors (PRRs), which are responsible for identifying the existence of microorganisms and act as the first line of defense against infection and tissue injury (25). PRRs are widely expressed and located not only on various immune cells including macrophages and dendritic cells but also on nonprofessional immune cells, such as cells of the neurovascular unit as well as cerebral vasculature, and even on the abnormal tumor cells (26). These conservative microbial production, including lipopolysaccharide, lipoteichoic acid, bacterial lipopeptides, peptidoglycan, and bacterial DNA, are commonly referred to PAMPs, and then stimulate PRRs, which eventually result in the migration of immune cells to the site of infection (27). However, in addition to the recognition of pathogens, the immune system is capable of responding to cellular damage, including acute organ rejection, systemic autoimmune diseases, and inflammatory diseases. Afterwards, Matzinger proposed the notion of endogenous danger signals that can sense harmful stimuli by activating the immune response following stress-induced damage (28). This type of inflammation can be triggered by danger-associated molecular patterns (DAMPs) (28) in response to stress and cell death, which is discriminative to PAMPs (**Table 1**). DAMPs, such as high mobility group box-1 protein (HMGB1), heat shock proteins (HSPs), uric acid, thioredoxin, galectins and so on, are released during oxidative stress or tissue damage and subsequently initiate an inflammatory response (27, 28). Despite PRRs are applied to detect PAMPs in order to further identify DAMPs, DAMPs are still viewed as menacing microbes (**Table 2**). In fact, the processes and mechanisms implicated in the necroinflammatory response are extremely complex. Cells dying by necrotic mechanisms, whether in a controlled manner or by accident, are characterized by cytoplasmic membrane damage, releasing their intracellular contents (e.g., DAMPs), which can be recognized by the immune system through signaling pathways, finally initiating a necroinflammatory process (29, 30). It has been hypothesized that the release of endogenous DAMPs can perform a vital function to evoke tissue inflammation and further excitation of regulatory cell death through autoamplification (31). Immune cells have the capability to detect various forms of hazardous cellular stresses and then transmit signals to elicit immune responses (32). Collectively, necroinflammation is associated with a persistent immune response and an inflammatory state, which induces the pathological process of human disease.

**Table 1:** Comparisons of PAMPs and DAMPs.

	PAMPs	DAMPs
Definitions	A particularly specific and highly conserved molecular structures owed by a certain types of microbial pathogens and their products, which can be recognized by nonspecific immune cells.	A type of molecular structures released into intercellular or blood circulation when the tissue or cells are suffering injury, hypoxia stress and then activated. It is the endogenous molecules released by organic cells.
Characteristics	(1) Specific to pathogenic microorganisms, but not produced by host cells.	(1) Activating innate immunity and adaptive immunity through variety of mechanisms.
	(2) Necessary for the survival or pathogenicity of microorganisms.	(2) Promoting the release of inflammatory mediators, regulating inflammatory response, and inducing the migration of immune cells to inflammatory sites.
	(3) As the molecular basis of extensively specific recognition of host innate immune cells.	(3) Increasing the ability of inflammatory cells to adhere and infiltrate.
Patterns	Lipopolysaccharide (LPS)	High mobility group box-1 protein (HMGB1)
	Lipoteichoic acid	Heat shock proteins (HSPs)
	Bacterial lipopeptides	S100 proteins
	Peptidoglycan	Uric acid
	Yeast and gram-positive bacteria	Adenosine and ATP
	Bacterial DNA	Galectins
	Flagellin	Thioredoxin
	Terminal mannose/fucose	IL-33/ST2

**Table 2:** DAMPs and PRRs that recognize menacing microbes.

DAMP	PRR
HMGB1	RAGE, TLR2/4
Serum amyloid A	TLR1/2
Fatty acids	TLR4
Hyaluronic acid	TLR2/4
Uric acid	NOD1/2, NLRP3
ATP	NLRP3, P2XR, P2YR, NOD1/2
snRNPs	RIG-I, MDA5,TLR7/8
dsDNA	TLR9, DAI, AIM2
Histone	TLR2/4/9, NLRP3
IC	FcR, TLR9
HSPs	TLR2/4
Surfactant Protein A/D	TLR2/4
Oxidized LDL	TLR4
Defensins	TLR2/4
RNA	TLR7
LL37	RAGE, TLR7/9
S100 proteins	RAGE, NOD1/2, TLR4
Reg111a	TLR4
Lactoferrin	TLR4

TLR, Toll-like receptor; NOD, nucleotide-binding oligomerization domain-containing protein; NLRP, NLR family, pyrin domain containing; ATP, adenosine triphosphate; snRNAP, small nuclear ribonucleoproteins; RIG-I, retinoic acid-inducible gene I; MDA, melanoma differentiation associated protein; DAI (ZBP1), dnadependent activator of interferon-regulatory factors; AIM2, absent in melanoma 2; HMGB1, high mobility group box-1 protein; RAGE, receptor for advanced glycation endproducts; IC, immune complex; FcR, Fc receptor; HSP, heat shock protein; LDL, low-density lipoprotein; LL37, cathelicidin antimicrobial peptide.

**Figure 3:**
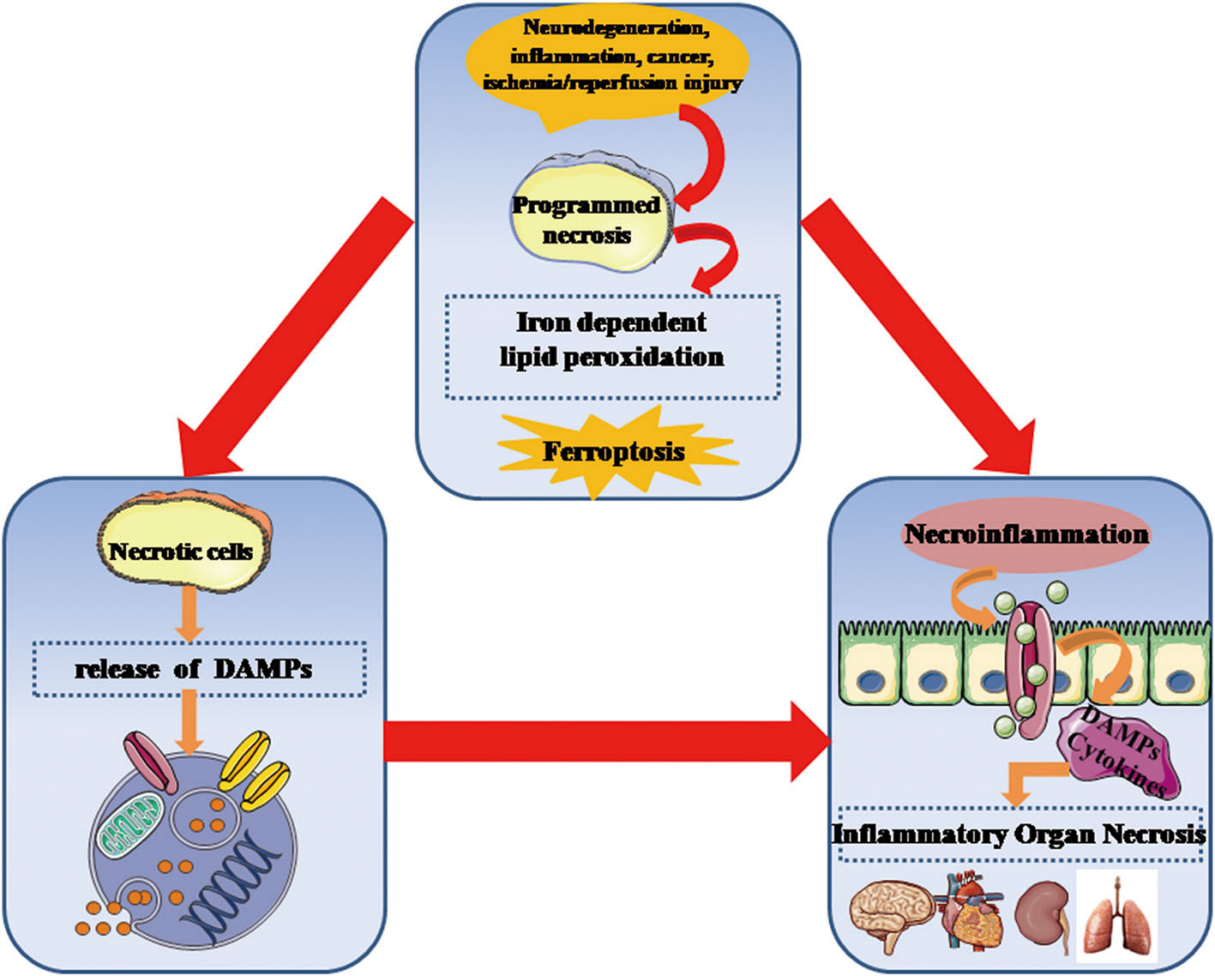
Ferroptosis is critically involved in the development of necroinflammatory diseases. Genetically programmed necrosis is initiated by variously systematic stress including oxidation, immunogenic molecules, metabolic disturbance, and ischemia/reperfusion injury. In the setting of oxidative stress, ferroptosis-induced lipid peroxidation and ROS can promote the programmed necrotic cells to release DAMPs as well as inflammatory cytokines that stimulate innate immune cells to enhance necroinflammatory response. DAMPs, danger-associated molecular patterns; ROS, reactive oxygen species.

It is accepted that ferroptosis, as a type of necrotic death, is more immunogenic than apoptosis to induce the release of inflammatory mediators and DAMPs, thus rendering the cellular environment highly proinflammatory state. Despite the release and function of DAMPs in ferroptotic cells remains unclear to a large extent, DAMPs can impact on initiating and perpetuating a necroinflammation during ferroptosis. A recent study has provided evidence that HMGB1 is a specific DAMP released by ferroptotic cells in an autophagy-dependent manner (33). Ferroptosis-induced inflammatory response appears to be significantly attenuated by intervention of anti-HMHB1 neutralizing antibodies, which indicates targeting HMGB1 release can effectively inhibit an necroinflammation in ferroptosis. Notably, the relationship between ferroptosis and the necroinflammatory response is outlined in **Figure 3**.

## Regulation of Ferroptosis in Necroinflammatory Response

### GPX4 Regulates Necroinflammation *via* Arachidonic Acid Metabolism During Ferroptosis

**Figure 4:**
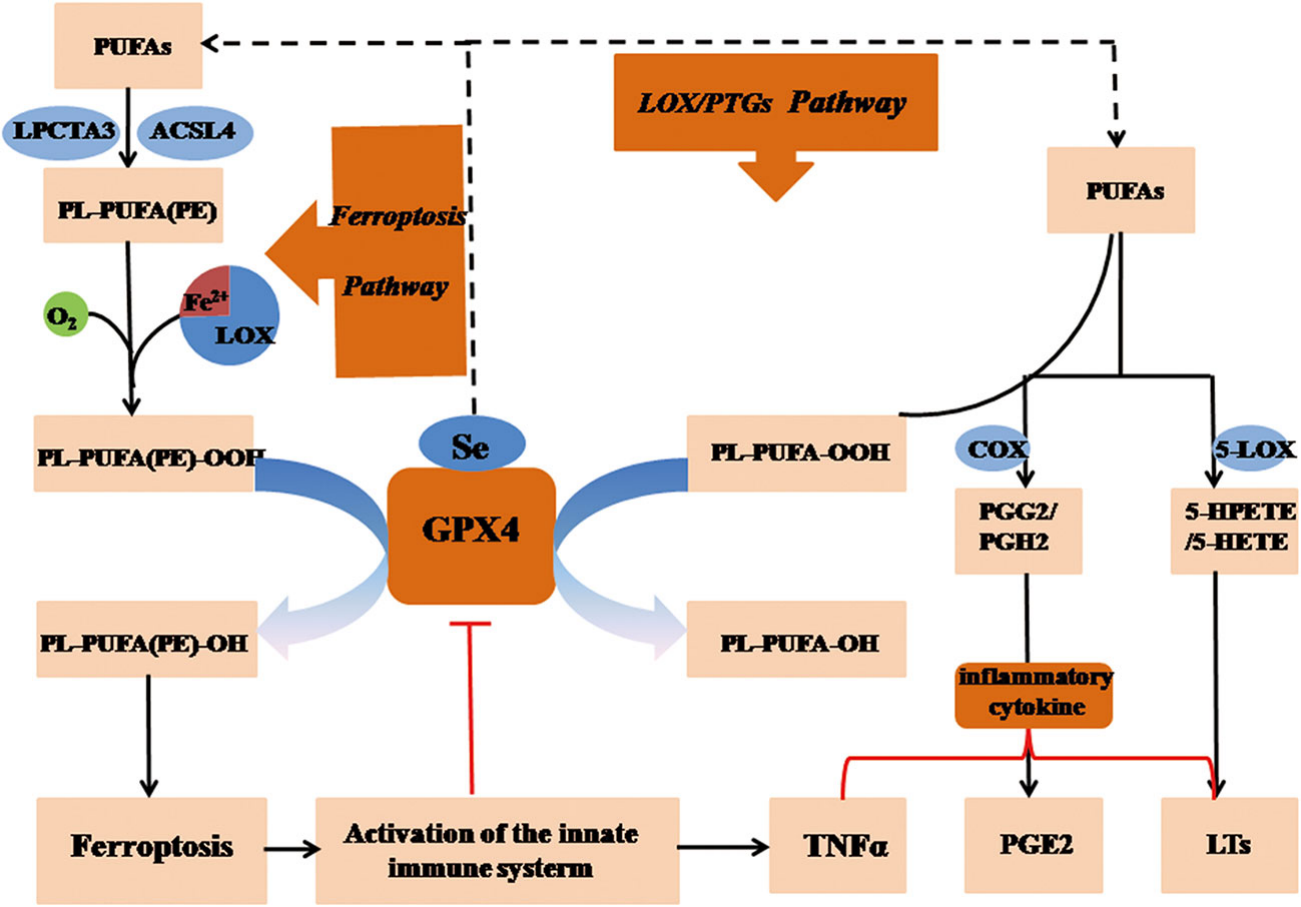
GPX4 regulates necroinflammation *via* arachidonic acid metabolism in ferroptosis. Peroxidized lipids and imbalanced metabolic arachidonic acid (AA) are comprised in ferroptotic process, which exerts an regulatory impact on the process of necroinflammatory response. Upregulation of GPX4 might results in a reduction in the cellular lipid hydroperoxide level, which inactivates PTGS and LOX, eventually inhibiting eicosanoid synthesis. The antioxidative enzyme GPX4 alleviates inflammatory response through eliminating oxidative materials produced in AA metabolism, and regulates the inflammatory state by modulating LOX and PTGS activity in ferroptosis. GSH, reduced glutathione; GSSG, oxidized glutathione; LOX, lipoxygenase; GPX4, glutathione peroxidase 4; H_2_O_2_, hydrogen peroxide; PGG_2_, prostaglandin G_2_; PTGS, prostaglandin-endoperoxide synthase; HPETE, hydroperoxyeicosatetraenoic acid; (P)LOOH, (phospho) lipid hydroperoxide; PUFA, polyunsaturated fatty acid; AA, arachidonic acid.

Iron-dependent peroxidized lipids and imbalanced metabolic arachidonic acid (AA) (13) are comprised in ferroptotic process, which exert regulatory effects on both occurrence and development of necroinflammatory diseases. However, in the early state of the ferroptotic-sensitization of cells, they might also play atypical roles in the mechanisms of over-activated autoimmune and innate immune system (34). Eicosanoids are derived from AA by the prostaglandin-endoperoxide synthase (PTGS) (35) or lipoxygenase (LOX) (36) enzymes, forming prostanoids, leukotrienes, respectively. Since these enzymes require lipid hydroperoxide for their activation, the overexpression of GPX4 results in a reduction in the cellular lipid hydroperoxide level, which effectively inactivates PTGS and LOX, eventually inhibiting eicosanoid synthesis (37–39). The antioxidative enzyme GPX4 alleviates inflammatory response through eliminating oxidative materials produced in AA metabolism, and regulates the inflammatory state by modulating LOX and PTGS activity for the duration of ferroptosis (40) (**Figure 4** **)**. The activities of LOX and PTGS are determined by the intracellular level of lipid peroxide for the reason that LOX consists of nonheme bound iron (Fe^2+^) while PTGS contains hemoglobin (Fe^3+^) in their corresponsive sites (41). Both LOX and PTGS are capable of promoting the catalysis of molecular oxygen during the oxidation of AA and other polyunsaturated fatty acids (PUFAs) (13) in a process. When suffering from oxidative stress, Fe^2+^ in LOX is oxidized to Fe^3+^, whereas Fe^3+^ is oxidized to a ferryl-oxo species, which immediately oxidizes the Tyr385, producing a tyrosyl radical in the LOX oxygenase active site (42).

#### The LOX Mechanism During Ferroptosis

Previous study showed LOX inhibitor induces GSH depletion which is now defined as ferroptosis (43). In addition, Seiler’s team reported that 12/15-LOX-defective cell was resistant to GSH depletion since tamoxifen-inducible GPX4 deficiency could be suppressed by 12/15-LOX specific inhibitors, and even result in cell death (44). They deduced that knockout of LOX family members could enhance ferroptosis, *via* cysteine/glutathione depletion and GPX inhibition. Overexpressing GPX4 in a neoplastic rat basophile cell line (RBL-2H3) strongly reduced the levels of leukotriene (LT) C4and LTB4, both products of the 5-LOX enzyme (44). This effect due to the reduction in 5-LOX activity instead of a drop in the rate of hydroperoxyeicosatetraenoic (HPETE) acid to hydroxyeicosatetraenoic (HETE) acid conversion (45).

LOX can regulate ferroptosis by generating LOX-derived proinflammatory metabolic products and stimulating the innate immune system. It seems reasonable that the function of GPX4 is impaired because of GSH depletion upon initial ferroptosis, which is the main cause of higher peroxidation in cells, and the eventual upregulation of LOX activity. On the basis of this mechanism, immune cells release proinflammatory mediators such as IL-6, γ-interferon, and tumor necrosis factor (TNF)-α, which might have an adverse influence on GPX4 activity (46). Ferroptotic cells not only trigger the immune response but can also release of DAMPs by means of self-degradation. Later, innate immune cells initiate LOX or PTGS enzymes, exacerbating inflammation by excreting LTs and hepoxilins. Taken together, activities of LOX and PTGS as well as DAMPs released by immune cells involve in ferroptotic mechanism in regulating necroinflammatory response.

#### The Role of PTGS in Ferroptosis

Expression of PTGS2 was found to be markedly upregulated in ferroptotic cells stimulated with Ras synthetic lethal 3 (RSL3) (7) or erastin, which confirmed the relationship between ferroptosis and necroinflammation (47). The PTGS2 inhibitor indomethacin, however, was incapable of preventing cells from undergoing ferroptosis, which was discovered in GPX4-defective cells (44, 48). Another study revealed that PTGS mRNA was similarly upregulated and prostaglandin E_2_ was simultaneously generated in the skin epithelium of GPX4-knockout mice. Celecoxib, an inhibitor of PTGS, destroyed hair follicle during hair morphogenesis in GPX4-knockout mice (49). The reason behind PTGS2 upregulation in ferroptotic cells is proposed to be linked to its role as a pharmacodynamic biomarker rather than the inhibitor of ferroptosis. Notably, PTGS inhibitors do not inhibit the occurrence of ferroptosis initiated by inducers or the genetic deletion of GPX4.

### GPX4 Regulates Necroinflammation by Preventing TNF-α-Mediated Reaction of the NF-κB Pathway

**Figure 5:**
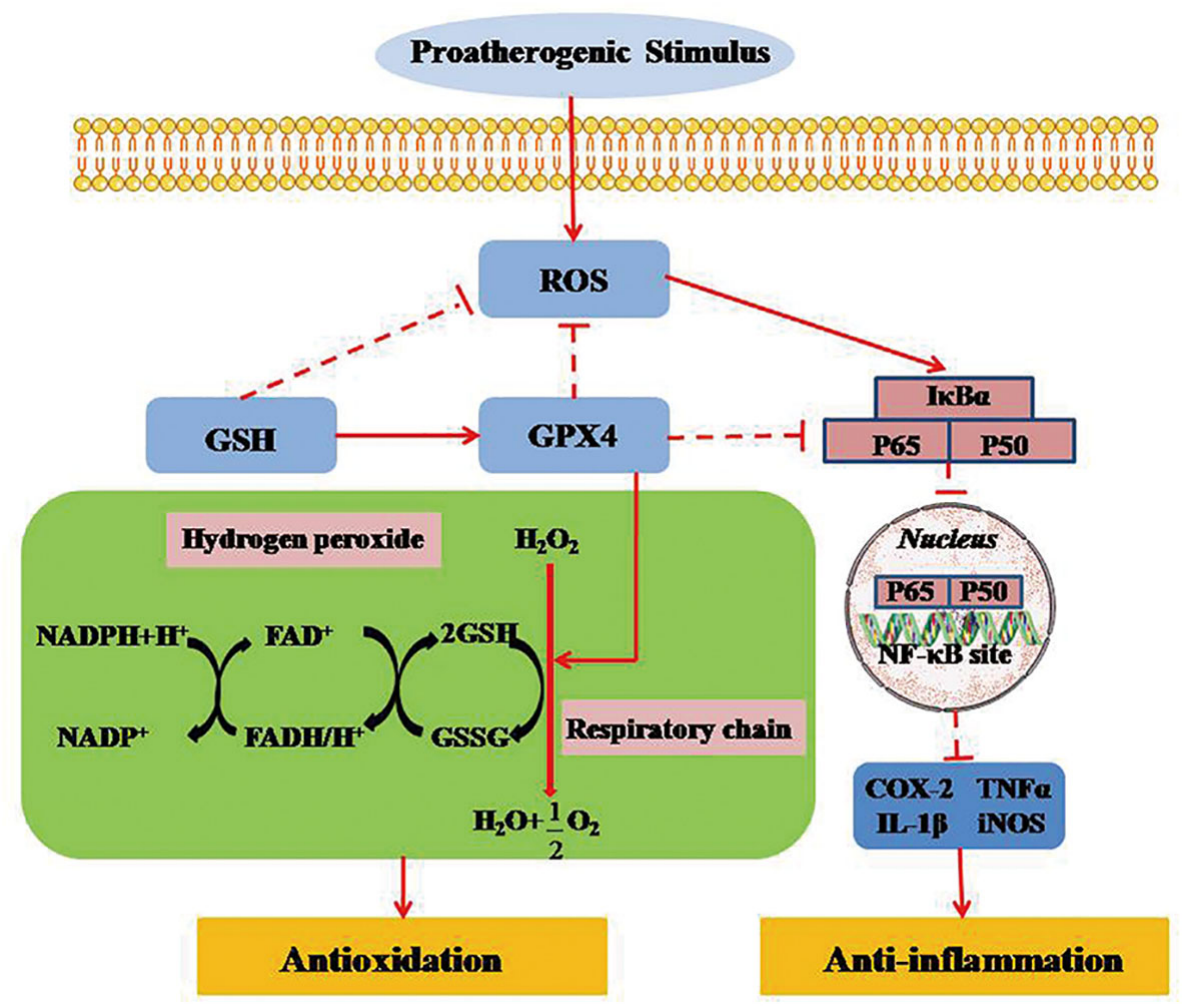
GPX4 regulates necroinflammation by inhibiting TNF-α-mediated NF-κB signaling. TNF-α regulates cell survival and death by activating the key transcription factor of NF-κB. NF-κB signal activation, as a downstream promoter element of TNF-α, is negatively suppressed in intracellular survival signaling. Inversely, TNF-α-mediated NF-κB signaling can be markedly activated when ROS is produced in mitochondria. GPX4 can attenuate the necroinflammatory response and suppress the inflammatory cytokines by down-regulating TNF-α-mediated NF-κB signaling pathway. ROS, reactive oxygen species; GPX4, glutathione peroxidase 4; GSH, glutathione; GSSG, glutathione disulfide; FAD/FADH, flavin adenine dinucletide; NADP/NADPH, nicotinamide adenine dinucleotide phosphate; NF-KB, nuclear factor κB; IκBα, inhibitor of NF-κB α; TNF-α, tumor necrosis factor-α; LT, leukotriene; COX, cyclooxygenase; NOS, nitric oxide synthase.

TNF-α is a proinflammatory cytokine, triggering cells a life-and-death struggle under the inflammatory and oxidative stress, which plays a vital role in the immune response and metabolic homeostasis (50). TNF can be identified by two types of receptors that TNFR1 (51) expresses on immune and endothelial cells and TNFR2 (51) regulates cell survival and death by activating a key transcription factor named nuclear factor (NF)-κB (52, 53). It is accepted that NF-κB protein function on tissue regeneration, cellular metabolism, as well as immune modulation to affect cellular fate and inflammatory progression (54, 55), and high level of ROS can activate NF-κB signaling downstream of TNF-α (56). Importantly, NF-κB signal activation, as a downstream promoter element of TNF-α, is negatively suppressed in intracellular survival signaling (57) (**Figure 5**). Inversely, TNF-α-mediated NF-κB signaling can be markedly activated when ROS is producted in mitochondria (58). Another research coincide with the view that ROS-generation in mitochondria could be inhibited in T cells by vitamin E, a mitochondria-specific antioxidant (59). Park and colleagues discovered that TNF-α, as an upstream molecule, might precisely activate the NF-κB signaling pathway and enhance the expression of NF-κB (60).

The NF-κB signaling pathway plays a vital role in the regulation of immune and inflammatory processes *via* the transcription of target genes (61). More studies indicate that selenoprotein family member of GPX4 can counteract hydroperoxide-modulated events by directly driving hydrogen peroxidation during the activation of NF-κB (62, 63). Indeed, GPX4 expressed on mammals has been shown to prevent the activation of TNF-α-mediated NF-κB signaling (64). Therefore, GPX4 could attenuate the necroinflammatory response and suppress the inflammatory cytokines by reducing the reaction of TNF-α-mediated NF-κB signaling pathway (65).

## Ferroptosis in Necroinflammatory Diseases

Increasing evidences substantiate the effect of ferroptosis on necroinflammatory diseases, such as neurodegeneration, I/R injury, inflammatory and immune diseases, transplant-related diseases as well as cancer. Theoretically, we speculate ferroptosis to be the potentially immunotherapeutic target in treatment of necroinflammatory diseases. Ferroptosis-induced tissues or organs on occurrence of necroinflammatory diseases are listed in **Table 3**.

**Table 3:** Ferroptotic tissues and organs presenting necroinflammation.

System diseases	Type of diseases	Evidences	References
Nervous system disease	Alzheimer’s disease	Deficiency of GPX4 resulted in reduction of neuron cells.	(66–68)
	Huntington’s disease	Multiple consumption of cystathioe γ-lyse (an enzyme for cysteine biosynthesis)	(69)
	Parkinson’s disease	Ferric sequestering agent showed protective effect.	(70–72)
Cardiovascular system disease	Ischemia/reperfusion (I/R) injury	GPX4 inhibited dysfunction of myocardial I/R area.	(73–77)
	Heart transplantation	Ferroptosis facilitated neutrophil recruitment in transplantation model.	(78)
Respiratory system disease	COPD induced by cigarette smoke (CS)	CS stimulated iron accumulation through NCOA4-mediated ferritinophagy.	(79, 80)
	Bronchial epithelial cell damage induced by Pseudomonas aeruginosa	Pseudomonas aeruginosa enhanced expression of pLoxA and oxidation.	(81, 82)
Digestive system disease	Inflammatory bowel disease (IBD)	Upregulating GPX4 and reversing NF-κB could relieve IBD.	(83–85)
	Nonalcoholic steatohepatitis	Hepatic ferroptosis initiated inflammation in steatohepatitis.	(86, 87)
Urinary system disease	Renal ischemia/reperfusion injury	I/R caused the release of proinflammatory mediators and aggravated cell death.	(88–92)
	Renal allograft rejection	Autoimmune and alloimmune kidney injury presented forms of necroinflammation and glomerulonephritis	(90)
	Acute kidney injury	Fer-1 prevented the necroinflammation of renal cells.	(93–95)
Integumentary system disease	Psoriasis	Downregulation of GPX4 resulted in ferroptosis and necroinflammation in psoriatic skin.	(96–98)
Genital system disease	Endometriosis	Dysregulated iron homeostasis induced endometriotic lesions with localized iron overload and inflammation.	(99–106)
	Male infertility	GPX4 variant led to impaired sperm development and male fertility.	(107–110)

### Diseases of the Nervous System

Ferroptosis involves in the pathogenesis of neurodegenerative diseases including Parkinson’s, Alzheimer’s, Huntington’s and neurodegeneration. In mouse models of the hippocampal region and the brain cortex, GPX4 knockout resulted in neuronal number reduction, lipid peroxidation, extracellular regulated protein kinase (ERK) activation, and inflammatory mediator release after administration with tamoxifen, which was enhanced by vitamin E deficiency whereas alleviated by the ferroptosis inhibitor of liproxstatin-1 (Lip-1) (66). The result revealed that cerebral cortex and hippocampus CA1 region seemed sensitive to ferroptosis. Interestingly, conditional GPX4 deficiency caused motor neuron degeneration, while GPX4 knockout resulted in a reduction of neuronal cells and inflammation of hippocampus (67, 68). Another model of Huntington showed cell death was suppressed by inhibiting lipid peroxidation when cells were pretreated with ferroptosis inhibitor of ferrostatin-1 (Fer-1) (111). Besides, cysteine synthetase depletion that caused necrotic cell death were detected in patients with Huntington’s disease (69). In Parkinson’s disease, it is demonstrated that protein kinase, defined as signal-regulated kinase-activating kinase (MEK), is independently activated by protein kinase Cα in extracellular, ultimately resulting in ferroptosis (70–72).

### Diseases of the Cardiovascular System

In I/R injury *in vivo*, overload iron plays a critical role in the ferroptosis of myocardial cells. It seems Fe^3+^ molecule activators to be more liable to induce ferroptosis in cardiomyocytes than RSL3, which is inversely relieved by iron chelator of deferoxamine (73). Meanwhile, glutaminase inhibitor is able to decrease infarct size, and it proves glutaminolysis to be closely associated with the pathophysiology of ferroptosis (74). On the other hand, GPX4 regarded as a mitochondrial targeted mutant causes the cell membrane incomplete and the creatine kinase decreased, which distinctly inhibits mitochondrial lipid peroxidation and the injury of myocardial cell in the I/R area (75). Furthermore, iron chelation could not only decrease the myocardial infarct size by reducing the release of serum myocardial makers, but also enhance the survival of doxorubicin-induced cardiac dysfunction by suppressing lipid peroxidation and mitochondrial iron load (76). In a mouse model of coronary artery ligation-induced I/R injury, pretreatment with Fer-1 reduced the intermediate production of hydroperoxy-arachidonoyl-phosphatidylethanolamine, and subsequently decreased the myocardial cell mortality (77).

### Diseases of the Respiratory System

Current studies on ferroptosis in pulmonary necroinflammatory disease mainly focus on chronic obstructive pulmonary disease (COPD) that is triggered by cigarette smoke (CS) and *Pseudomonas aeruginosa*-induced damage in bronchial epithelial cells. It was reported that CS not only induced necroptosis but also promoted the release of DAMPs in epithelial cells, thereby contributing to airway necroinflammation (79, 80). In the model of COPD established by exposure to CS, down-expression of GPX4 might ultimately induce iron accumulation and lipid peroxidation, which confirmed the key effect of ferroptosis on CS-induced COPD and further revealed the mechanism of iron accumulation inducing ferritinophagy mediated by NCOA4 in epithelial cells (81). As is revealed in human bronchial epithelial (HBE) cells invaded by pseudomonas aeruginosa, the feature of ferroptosis presented that polyunsaturated fatty acids was oxidized by pLoxA into 15-HOO-AA-PE (82).

### Diseases of the Digestive System

Growing studies have indicated that ferroptotic mechanisms regulate necroinflammatory diseases of the digestive system including Crohn’s disease (CD), inflammatory bowel disease (IBD), ulcerative colitis (UC) and nonalcoholic steatohepatitis. IBD is an intestinal dysfunction induced by chronic inflammation, featured in the reduction of crypt, villus atrophy as well as inflammation of the intestinal mucosa and submucosal tissues induced by neutrophil accumulation (112–114). It generally occurs in the settings of gene mutation, oxidative stress, traumatic stress, relocation stress syndrome or environmental factors. Oxidative stress is considered as the main pathological process associated with ferroptosis, and is deemed to be the major factor in the prognosis of necroinflammation in IBD (115, 116). Emerging researches emphasis on the relationship between iron metabolism, intestinal microecological health, and intestinal inflammatory diseases. It has been proven that excessive iron induces IBD, triggering oxidative stress and even cell death (117). On account of the damage of intestinal integrity induced by excessive iron, oxidative reactions may destruct the physical barrier composed of intestinal mucosal epithelial cells leading to intestinal dysfunction (118). Accordingly, a novel inhibitor of inflammation and oxidation called pyrrolidine dithiocarbamate is demonstrated to be effective on improving IBD symptoms by upregulating GPX4 (83–85), which inversely suppressed NF-κB signaling (119). With respect to CD, GPX4 on epithelium is found to be down-expressed, which leads to intestinal function and even ferroptosis (120, 121). In order to further explore the function of GPX4 in intestinal epithelial cells, Sander et al. structured *Gpx4^−/−^* intestinal epithelial MODE-K cells with the specific GPX4^-^ small-interfering RNA (siGPX4) to silence GPX4 expression. It was resulted that intestinal epithelial cells with inactive GPX4 were more sensitive to ferroptosis, and multiple of necroinflammatory cytokines including IL-6, IL-12, IFN-γ and TNF-α were detected in supernatant (122, 123).

Recent studies indicate that ferroptosis-induced necroinflammation is relevant to nonalcoholic steatohepatitis by triggering cytokine release (86, 87). A research on nonalcoholic steatohepatitis in a murine model demonstrated that ferroptosis inhibitor effectively suppressed hepatic cell death through the inhibition of immune cell function and migration. Therefore, ferroptosis in hepatic cells might serve as a therapeutic target for the treatment of necroinflammation in nonalcoholic steatohepatitis.

### Urinary System Diseases

Necroinflammation in renal I/R injury dependent on a transient insufficient blood supply finally results in critical damage in renal cells (124). Similarly, renal-necrosis is generally associated with autoimmune kidney injury, which is caused by various types of glomerulonephritis (88), interstitial nephritis (89), and allograft rejections (90). When renal tissue or cells undergo ischemic stress, endogenous DAMPs together with intracellular proinflammatory mediators, such as HSP, HMGB1, metabolites, and gene fragments, are largely release, stimulating immune cells to result in inflammatory cascade (91, 92). Immune cells including T cells and macrophages are rapidly activated to stimulate neutrophil aggregation, and release cytokines of IL-6, IL-12, IL-1α, and TNF-α to promote the migration of antigen presenting cells (e.g.dendritic cells) (125, 126). A continuous auto-amplification is formed when high level of mediators irritate cell death program. It follows that necrocytosis induces immunosuppression, and organ failure occurs (127).

Necroinflammation is regulated by ferroptosis in acute kidney injury. Preliminary studies indicated that Fer-1 could not only attenuate I/R injury-induced tubular injury but also decreased serum creatinine and urea nitrogen level (93). Pretreatment with Fer-1 was able to protect renal proximal tubular cells from necrosis in the renal ischemic tissue area by decreasing ROS levels (94). SLC7A11 is shown to be negatively regulated by the mutant cancer suppressor p53 (3KR) gene that down-regulates Xc- system to promote ferroptosis (95). Silencing p53 mutation results in SLC7A11 overactivation and protective effect on renal cells from ferroptotic injury. In accordance with the above studies, tubular cell with p53 deficiency is resistant to ferroptosis induced by acute kidney injury (128, 129). It is the reason that tubular cell with GPX4 deficiency is prone to suffering from ferroptotic necrosis and high mortality rates in mice (10).

### Integumentary System Diseases

GPX4 knockout has been proved to induce ferroptosis-related necroinflammation in skin tissue (130–132). For the reason that ceramide analogs are testified to be an effective therapeutic in animal experiments, it is speculated that upregulating GPX4 might significantly relieve skin from ferroptosis-induced necroinflammatory injury (96, 97). In order to further investigate the interactive mechanism between GPX4 inactivation, ferroptosis and necroinflammation, Arbiser and colleagues collected data from healthy skin samples and psoriatic skin samples to analyze genetic sequences. They found necroinflammation in psoriatic skin samples, and treatment with ferroptosis inducer that inhibited Xc- system activity could decrease the level of Nrf2 (98). These findings provide a novel viewpoint on intrinsic connection between ferroptosis and necroinflammation in skin disease.

### Diseases of the Reproductive System

Ferroptosis is reported to involve in reproductive necroinflammatory processes, such as endometriosis and male infertility (e.g., oligospermia). Selenium that is applied to the GPX4 synthesis plays an essential role in male fertility and sperm development (99, 100). GPX4 maintains sperm stability by acting as a major structural protein of the mitochondria capsule in the central part of mature spermatozoa. A recent clinical study demonstrated that approximately 30% oligoasthenozoospermia in infertile men showed a down-regulation of GPX4 when compared to healthy men with normal testes and spermatozoa (101). Another research revealed that sperm vitality obviously declined when GPX4 inactivated or dysfunctioned, which confirmed the key role in spermatogenesis and the process of embryo development in mice (102). Moreover, clinical investigations demonstrated Nrf2 was involved in the regulation of ferroptosis in oligospermia (103, 104). In comparison to the control group, Nrf2 suppressing ferroptosis was notably down-regulated in mice with oligospermia (105, 106).

Studies on female reproductive system and endometriosis showed that overload iron was the major cause for endometriotic lesion (107–109). Ferroptosis inhibitor of deferoxamine cannot reverse pathological tissue damage, but improve iron metabolism, as well as reduce the proliferation of macrophages, which finally alleviates inflammatory response (110). Inversely, endometriotic lesion results in iron and ROS accumulation, which obstructs the normal growth of ectopic endometrial cells (133–135).

### Transplantation-Related Diseases

Transplantation is currently becoming a life-saving straw for critical patients with organ function failure. However, graft rejection remains the mainly negative impact on the long-term prognosis due to an interaction between innate and adaptive immune responses. Increasing evidences document that necroinflammation is regarded as the essential pathological process in graft injury, and ferroptosis is pyramidally being proved to relate to transplantation-induced necroinflammatory response.

Previous studies reported that ferroptosis induced neutrophil migration and adhesion to the vascular endothelial cells by TLR4/Trif/type I IFN-dependent signaling pathway, in turn leading to heart transplantation-related injury (78). Accordingly, pretreatment of myocardial cells with Fer-1 could effectively inhibit neutrophil recruiting in the early stage of heart transplantation (78). These studies indicate that ferroptosis amplifies a cycle between sterile inflammation and immunological rejection, and aggravates graft injury. In the liver transplantation, inevitably hepatic I/R injury during the process of organ procurement may cause primary nonfiction and urgent rejective injury in the graft liver (136). Lipid peroxidation, upregulation of a ferroptotic biomarker Ptgs2 (136) as well as liver injury are shown in the mutine model of liver transplantation. High level of serum ferritin, a sign of iron load in ferroptosis, is also detected, which can be inhibited by Fer-1 (137). Moreover, a recent study on islet transplantation was consistent with the abovementioned viewpoint that ferroptosis involved in oxidative injury and necroinflammation after islet transplantation. The viability of transplanted islet was evaluated by lactate dehydrogenase (LDH), and outcomes revealed that pretreatment islet with ferroptosis inhibitors Fer-1 or desferrioxamine (DFO) improved graft injury in an immunodeficient mouse transplant model (138).

### Cancer

Ferroptosis was first discovered in tumor cells when explored the death manner induced by lethal RAS-mutant gene, and it could be induced in most types of tumor by FINs erastin and RSL (5, 7). Erastin of 117 cancer cell lines from various tissues were detected to study RAS mutant mediated ferroptosis, and it was noted that kidney cancer cells showed the most sensitive to erastin (5). In the subsequent studies, they demonstrated that erastin, as ferroptosis inhibitor, contributed to improving pesticide effect of oncology chemotherapy, and was applied to test the sensitivity of cancer to ferroptosis (139).

On account of ROS being essential for existence and proliferation, cancer cells are invariably dependent on intracellular GSH. When authigenic cysteine is consumed to produce ROS, resulting in deficient for GSH synthesis, more extracellular cysteine need to be transferred by system Xc-. Therefore, disposition cancer cells with a recombinant cyste inase enzyme leading to exhaustion of cysteine, might selectively induce cell death in cancer cells (140). Of note, it is hypothesized that protein p53 possibly relates to system Xc- activity due to the cause that inase inhibitor sorafenib acting on system Xc- and regulated by p53 can induce ferroptosis (23).

Many studies have suggested that cancer cells with manifestation of treatment-resistant high-mesenchymal cell state greatly depend on lipid metabolic enzymes which are relevant to the ferroptotic signaling pathway. Thus, these cancer cells display more sensitive and vulnerable to ferroptosis in the setting of GPX4 inactivation. Similarly, a recent study supported this standpoint that the viability of clear-cell renal cell carcinomas (ccRCC) significantly decreased, which showed a hypersensitivity to GPX4 silencing and vulnerability to ferroptosis (141). Importantly, reduced fatty acid peroxidation owning to the inhibition of β-oxidation can effectively interdict ccRCC growth by suppressing ferroptosis. These results indicate that targeting ferroptosis contributes to exploring a novel therapy for overcoming drug resistance in cancer.

## Conclusions

Ferroptosis is a novel-proposed regulated cell death process that relies on overload iron and glutathione metabolism, and plays a regulated role in necroinflammatory diseases. It is confirmed that ferroptosis involves in the pathologic process of various necroinflammatory diseases and regulates necrotic cell death. Recently, mechanism of ferroptosis in necroinflammatory diseases is being continually explored in infratest and many progresses have been made. However, certain limitations remain to be overcome. Firstly, comparing to our in-depth understanding of mechanisms involved in classical cell death programs, we know little about the mechanism of ferroptosis. Despite the roles of lipid peroxidation and inflammation are relatively well documented, more precise signaling pathways that may regulate the necroinflammatory response in relation to ferroptosis seem not clear. Second, potential ability of ferroptosis to activate the innate immune system to release inflammatory mediators and generate an immune response remains to be further explored. Third, most of the ferroptosis-related researches to date have depended on the established animal models, which have their own restrictions. Thus, conducting these hypotheses in clinical trials will be more reasonable. Furthermore, perspective researches should emphasize on the regulated mechanism of ferroptosis mediated by upstream and downstream signaling molecules as well as the intermolecular interactions. Hence, targeting ferroptosis might provide a potential therapy for necroinflammatory diseases in the future.

## Author Contributions 

J-yL conducted the literature review and drafted the manuscript, which Y-mY and Y-pT conceptualized, supervised and revised. All authors contributed to the article and approved the submitted version.

## Funding

This work was supported by grants from the National Natural Science Foundation of China (81730057, 81873946) and the National Key Research and Development Program of China (2017YFC1103302).

## Conflict of Interest

The authors declare that the research was conducted in the absence of any commercial or financial relationships that could be construed as a potential conflict of interest.

## Publisher’s Note

All claims expressed in this article are solely those of the authors and do not necessarily represent those of their affiliated organizations, or those of the publisher, the editors and the reviewers. Any product that may be evaluated in this article, or claim that may be made by its manufacturer, is not guaranteed or endorsed by the publisher.

## Abbreviations

ACSL4, long-chain-fatty-acidacetyl-Coenzyme A synthase 4; GPX4, glutathione peroxidase 4; ROS, reactive oxygen species; DAMPs, danger-associated molecular patterns; PCD, programmed cell death; NPCD, non-programmed cell death; GSH, glutathione; LOX, lipoxygenase; PAMPs, pathogen-associated molecular patterns; PRRs, pattern recognition receptors; AA, arachidonic acid; PTGS, prostaglandin-endoperoxide synthase; HPETE, hydroperoxyeicosatetraenotic acid; HETE, hydroxyeicosatetraenoic acid; PUFAs, polyunsaturated fatty acids; ILs, interleukins; TNF-α, tumor necrosis factor-α; LT, leukotriene; NF-κB, nuclear factor κB; I/R, ischemia/reperfusion; RSL3, Ras synthetic lethal 3; Fer-1, ferrostatin-1; COPD, chronic obstructive pulmonary disease; CS, cigarette smoke; HBE, human bronchial epithelial; IBD, inflammatory bowel disease; UC, ulcerative colitis; CD, Crohn’s disease; IECs, intestinal epithelial cells; AKI, acute kidney injury; HMGB1, high mobility group box-1 protein. HSF, heat shock factor; HSPB1, heat shock factor1; SLC7A11, transmembrane protein transporter solute carrier family 7 member 11; SLC3A2, transmembrane regulatory protein solute carrier family 3 member 2; PE, phosphatidylethanolamine; AdA, adrenaline; Sp1, special protein 1; AIFM2/FSP1, apoptosis-inducing factor mitochondria-associated 2; CoQ, coenzyme Q; NADP, nicotinamide-adenine dinucleotide phosphate; NCOA4, nuclear receptor coactivator 4; IREB2, iron responsive element-binding protein 2; Atg5, autophagy-related 5; Atg7, autophagy-related 7; Nrf2, nuclear factor erythroid 2-related factor 2; Keap1, Kelch-like ECH-associated protein 1; HO-1, home oxygenase-1; FTH1, ferritin heavy chain; HSPs, heat shock proteins; LDH, lactate dehydrogenase; DFO, desferrioxamine; ccRCC, clear-cell renal cell carcinomas.
